# Related Factors of Patent Ductus Arteriosus in Preterm Infants: A Systematic Review and Meta-Analysis

**DOI:** 10.3389/fped.2020.605879

**Published:** 2021-01-05

**Authors:** Chang Liu, Xingwang Zhu, Dinggang Li, Yuan Shi

**Affiliations:** ^1^Department of Neonatology, Children's Hospital of Chongqing Medical University, Chongqing, China; ^2^National Clinical Research Center for Child Health and Disorders, Chongqing, China; ^3^Ministry of Education Key Laboratory of Child Development and Disorders, Chongqing, China; ^4^Chongqing Key Laboratory of Pediatrics, Chongqing, China

**Keywords:** patent ductus arteriosus, preterm infants, related factors, systematic review, meta-analysis

## Abstract

**Background:** Patent ductus arteriosus (PDA) is a dramatically harmful disease in the neonatal period, in particular common in preterm infants, and our study was to determine related factors of PDA in preterm infants.

**Methods:** A comprehensive literature review was conducted in PubMed, EMBASE, and Web of Science. The pooled odds ratio and standard mean difference were calculated to compare dichotomous and continuous variables, respectively. In addition, we also assessed the heterogeneity and publication bias and carried out sensitivity analysis for each related factor.

**Results:** We included 45 studies with 87,419 individuals. After the primary analysis and a series of adjustments, results showed chorioamnionitis, lower gestational age, lower birth weight, bronchopulmonary dysplasia, intraventricular hemorrhage, necrotizing enterocolitis, respiratory distress syndrome, sepsis, surfactant treatment, ventilation, and lower platelet count had a positive correlation with PDA, while small for gestational age decreased the incidence of PDA in preterm infants. Besides, premature rupture of membranes, preeclampsia, antenatal steroids, male gender, mean platelet volume, and platelet distribution width were found to have no statistically significant relationship with PDA.

**Conclusion:** Preterm infants with more immature characteristics generally have a higher likelihood to develop PDA. The prevention, diagnosis, and management of PDA may depend on these results, and effective measures can be taken accordingly.

## Introduction

Ductus arteriosus (DA), an essential component of the circulatory system during fetal life, connecting the main pulmonary artery to the descending aorta, diverts ventricular output away from the lungs and toward the placenta *in utero* (1, 2). At term, the spontaneous functional closure of DA normally occurs within 24 to 48 h after delivery due to an increase in partial arterial oxygen tension and a decrease in circulating prostaglandins and prostaglandin receptors in the DA's wall. Generated by “hypoxic zone,” the DA will anatomically close within the next 2 to 3 weeks (1–4). Nevertheless, the DA sometimes fails to close, leading to a condition called patent ductus arteriosus (PDA), which affects 57 of every 100,000 full-term neonates (2). Furthermore, failure to close is strikingly more common in preterm infants (5), and it is estimated that approximately 65% of infants <28 weeks still have an unclosed DA by day 7 after birth (6).

Not only does PDA have a rather high morbidity in preterm infants, but also it has a high severity. The hemodynamic consequence of PDA is a left-to-right shunt (5), resulting in a combination of pulmonary overcirculation and reduced organ perfusion (1, 3–5), which will further entail a series of harmful clinical outcomes such as intraventricular hemorrhage (IVH), bronchopulmonary dysplasia (BPD), necrotizing enterocolitis (NEC), feeding intolerance, decreased glomerular filtration rate, and even mortality (1, 4, 5, 7–10). Therefore, PDA is one of the major causes of long hospitalization duration and poor prognosis for preterm infants and has a seriously damaging effect on the growth and development of newborns, on account of which the early diagnosis and management of PDA are definitely essential. Taking the above into consideration, identifying related factors clearly is worthy of more attention because it can provide numerous helpful insights to develop diagnosis, management, and treatment consensuses to prevent PDA for preterm infants.

A growing body of evidence from recent articles has suggested that a few variables have a causal impact on PDA, notwithstanding some of which are still controversial (11–19). Although there have been several meta-analyses endeavoring to address these conflicting disputes, unfortunately, they focus only on certain one related factor of PDA (20–23), while it is a multifactorial disease, and many factors play different roles in the pathophysiological process. Accordingly, there is an increasingly urgent need to summarize a comprehensive conclusion containing multiple related factors at the same time, and the present study aims to resolve the issue and to evaluate as many potential related factors as possible for PDA.

## Methods

### Search Strategy

We adhered to the recommendations made by the Preferred Reporting Items for Systematic Reviews and Meta-Analyses (PRISMA) guidelines (24). The study was registered in PROSPERO, number CRD42020171459. PubMed, EMBASE, and Web of Science were systematically searched until March 2020, and only those studies written in English or Chinese were included. The search strategy used in PubMed is shown in Table 1, and similar search strategies were also taken in EMBASE and Web of Science.

**Table 1:** PubMed search strategy.

**No**.	**Search items**
#1	((((((((((Factor, Risk [Title/Abstract]) OR Factors, Risk [Title/Abstract]) OR Risk Factor [Title/Abstract]) OR Factors [Title/Abstract]) OR Factor [Title/Abstract])) OR relationship [Title/Abstract])) OR “Risk Factors” [Mesh]))
#2	(((((((((((((((Infants, Premature[Title/Abstract]) OR Preterm Infant[Title/Abstract]) OR Premature Infant[Title/Abstract]) OR Preterm Infants[Title/Abstract]) OR Infant, Preterm[Title/Abstract]) OR Infants, Preterm[Title/Abstract]) OR Premature Infants[Title/Abstract]) OR Neonatal Prematurity[Title/Abstract]) OR Prematurity, Neonatal[Title/Abstract])) OR “Infant, Premature”[Mesh]) OR (((((((Neonates[Title/Abstract]) OR Infants, Newborn[Title/Abstract]) OR Newborn Infant[Title/Abstract]) OR Newborn Infants[Title/Abstract]) OR Newborns[Title/Abstract]) OR Newborn[Title/Abstract]) OR Neonate[Title/Abstract])) OR “Infant, Newborn”[Mesh]))
#3	((“Ductus Arteriosus, Patent”[Mesh]) OR (((((Patency of the Ductus Arteriosus[Title/Abstract]) OR Patent Ductus Arteriosus[Title/Abstract]) OR Patent Ductus Arteriosus Familial[Title/Abstract]) OR PDA[Title/Abstract]) OR ductus arteriosus[Title/Abstract])))
#4	#1 AND #2 AND #3

*PDA, patent ductus arteriosus*.

### Study Selection

**Figure 1:**
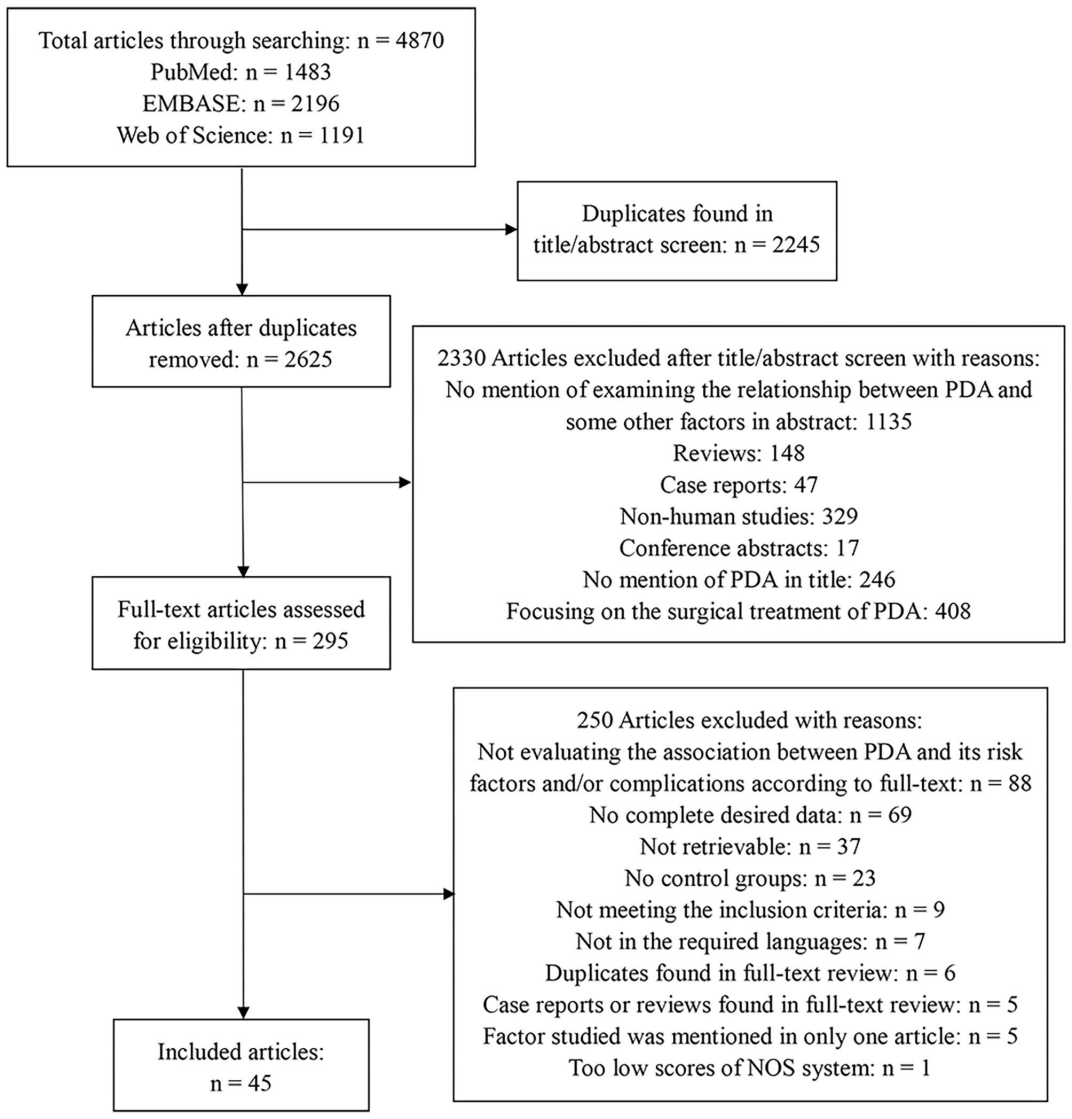
Flow diagram describing the systematic review of related factors of patent ductus arteriosus in preterm infants.

We included eligible studies that compared infants with PDA to infants without PDA. Articles were included if they belonged to case-control studies, cohort studies, or retrospective cohort studies. We excluded studies if they enrolled infants with gestational age (GA) ≥37 weeks or/and birth weight (BW) ≥2,500 g. Meanwhile, studies without control group data were excluded, too. Two reviewers (C.L. and X.Z.) independently assessed the titles and abstracts of studies that we found through the search strategies. Then, the full texts were retrieved, and the eligibility according to the inclusion criteria was evaluated. Any disagreement was resolved through a discussion with a third reviewer (Y.S.). The PRISMA flow diagram of the search is shown in Figure 1.

### Data Extraction and Quality Assessment

The following information was extracted from each study: name of the first author, publication year, country/region, study type (case-control or cohort, prospective or retrospective), the number of included infants, mean BW and GA of included infants, definition of PDA, the time of PDA assessment after birth, and aim of study, which is illustrated in Supplementary Table 1. Then, we analyzed these related factors that were mentioned in at least three articles, and both adjusted and unadjusted results were compiled and compared if included articles could provide sufficient data.

The Newcastle–Ottawa Scale (25) was applied to observational studies to review the risk of bias. We evaluated included studies by scoring them, with a maximum of nine points, consisting of four points in selection of the study groups, two points in comparability of the groups, and three points in assessment of outcome/exposure. The detailed results are displayed in Supplementary Table 2.

### Data Synthesis and Analysis

We combined and analyzed studies using Comprehensive Meta-Analysis v. 3.0 software. The pooled odds ratio (OR) and standard mean difference (SMD) were calculated to compare dichotomous and continuous variables, respectively. The method of Wan et al. was used to estimate the mean and standard deviation when continuous variables were reported as median and range/interquartile range in studies (26). The *I*^2^ statistic and *P*-value of heterogeneity test were used for testing the heterogeneity. Once there was no significant heterogeneity (*P* > 0.10, *I*^2^ < 50%), the fixed-effects model was adopted; otherwise, the random-effects model was applied. We also analyzed publication bias when more than 10 studies were available for one related factor (27). The publication bias was assessed through funnel plot and Egger's test. When *P* < 0.10 in Egger's test, we considered it had a significant publication bias and used trim-and-fill computation to adjust the result. Sensitivity analysis was performed by removing the articles one by one, then we compared the ORs (SMDs) before and after the removal. When the OR (SMD) changed significantly, we excluded that article in final analysis.

## Results

### Literature Search

The electronic database search yielded 4,870 articles in total. After removing 2,245 duplicates, we examined 2,625 article titles and abstracts. After independent review by two reviewers, 2,330 studies were deemed not relevant to related factors of PDA in preterm infants. The full-text versions were evaluated for the remaining 295 articles, of which 250 were excluded because of various reasons. Finally, we included 45 studies, and the flow diagram summarizing the study identification and selection is shown in Figure 1.

### Characteristics of Included Studies

The baseline characteristics of the 45 studies representing a total of 87,419 preterm infants are shown in Supplementary Table 1. Among the 45 studies, 25 were retrospective studies, 18 were prospective studies, and two had unclear designs. These 45 included studies comprised 34 cohort studies and 11 case-control studies. The sample sizes ranged from 31 to 43,576 participants, with mean GA ranging from 25.7 to 31.1 weeks and mean BW ranging from 761 to 1,852 g. The definitions of PDA in each study varied significantly, from hemodynamically significant PDA to echocardiogram or whether receiving treatment or not. The time of PDA assessment and research aims of each study were also markedly different and the concrete details are listed in Supplementary Table 1.

### Quality Assessment

The methodological quality of the enrolled studies was satisfactorily high, with 13 studies scoring 9 of 9 points on the Newcastle–Ottawa Scale, 4 scoring 8, 23 scoring 7, and 5 scoring 6, which is the minimum score of eligibility, and the reasons for downgrade of each study are presented in Supplementary Table 2. However, one article was excluded because it scored only five points in quality assessment process, as described in Figure 1.

### Maternal Factors

**Figure 2:**
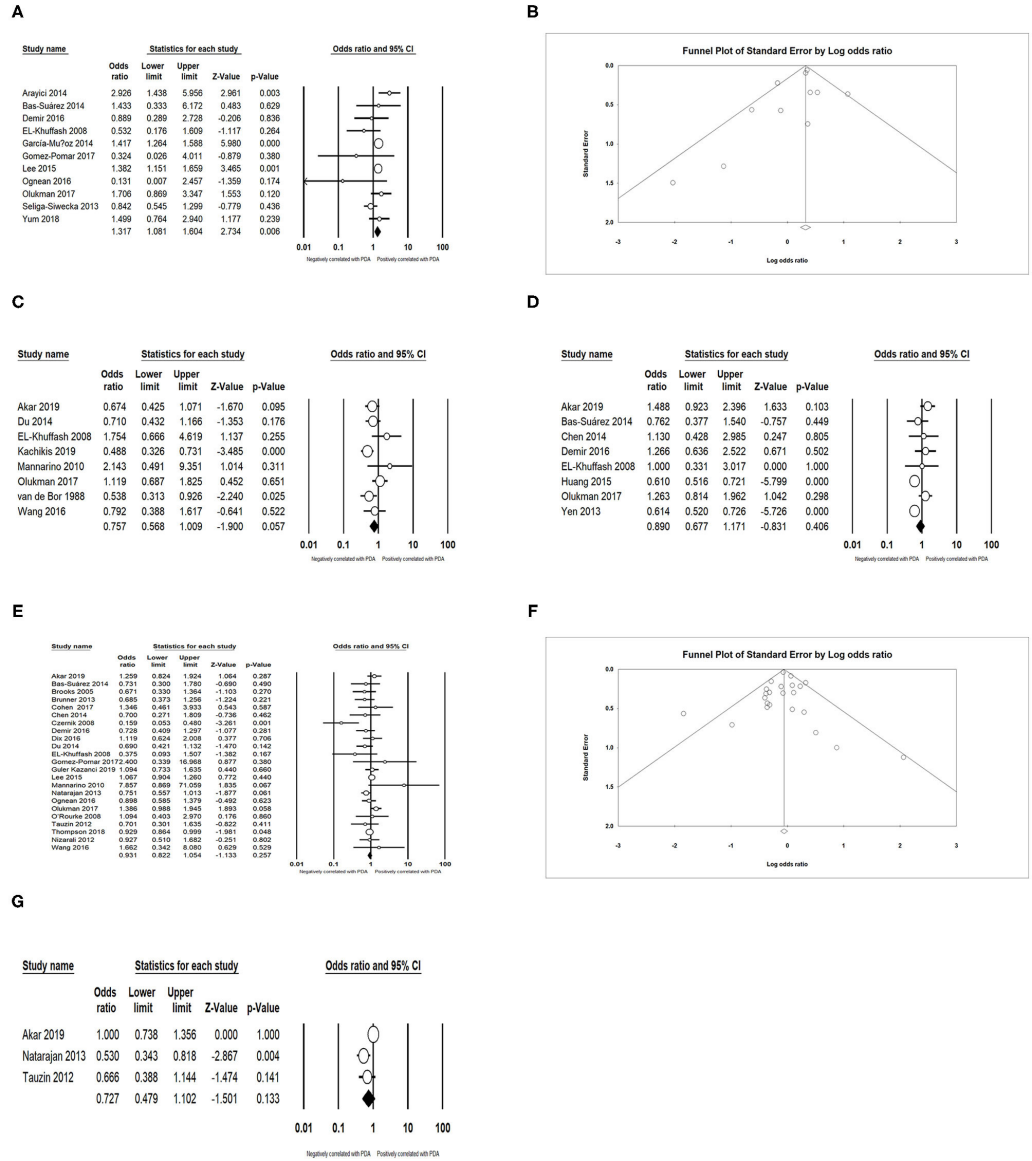
Results of maternal factors in primary analysis. **(A)** Forest plot of association between PDA and chorioamnionitis; **(B)** funnel plot for publication bias of the association between PDA and chorioamnionitis; **(C)** forest plot of association between PDA and premature rupture of membranes; **(D)** forest plot of association between PDA and preeclampsia; **(E)** forest plot of association between PDA and antenatal steroids (according to unadjusted data); **(F)** funnel plot for publication bias of the association between PDA and antenatal steroids (according to unadjusted data); **(G)** forest plot of association between PDA and antenatal steroids (according to adjusted data). PDA, patent ductus arteriosus.

In the primary analysis, infants whose mothers were diagnosed with chorioamnionitis (CA) were more likely to develop PDA than those whose mothers were not diagnosed with CA [OR = 1.317, 95% confidence interval (CI) = 1.081–1.604; *I*^2^ = 41.578%] (Figures 2A,B). We did not find any significant association between PDA and premature rupture of membranes (PROM) (OR = 0.757; 95% CI = 0.568–1.009; *I*^2^ = 47.358%) (Figure 2C) and preeclampsia (OR = 0.890; 95% CI = 0.677–1.171; *I*^2^ = 72.730%) (Figure 2D). There was also no association between PDA and antenatal steroids according to both pooled unadjusted data (OR = 0.931; 95% CI = 0.822–1.054; *I*^2^ = 36.655%) (Figures 2E,F) and adjusted data (OR = 0.727; 95% CI = 0.479–1.102; *I*^2^ = 66.286%) (Figure 2G).

### Neonatal Characteristics

**Figure 3:**
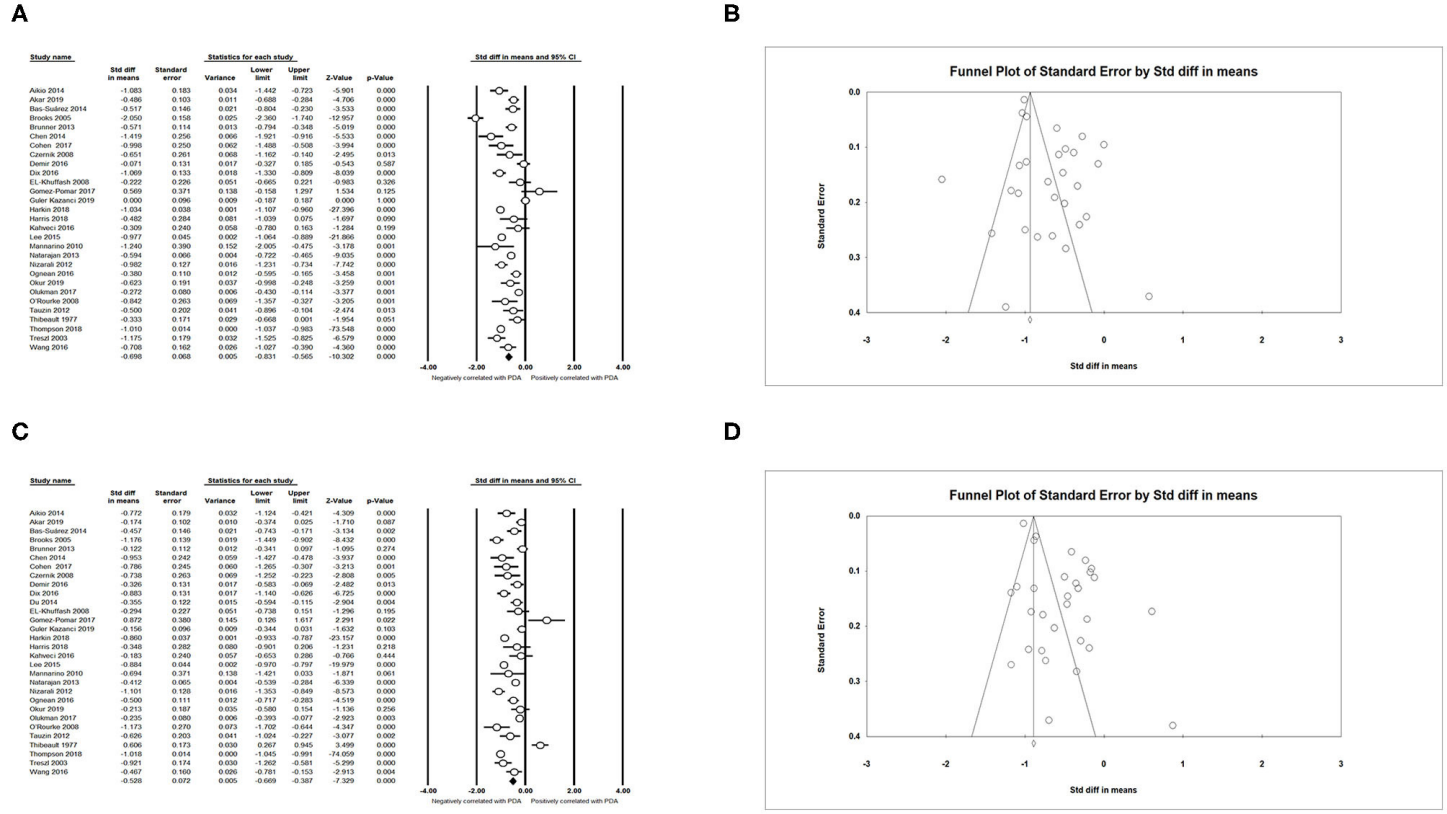
Results of neonatal characteristics in primary analysis. **(A)** Forest plot of association between PDA and gestational age; **(B)** funnel plot for publication bias of the association between PDA and gestational age; **(C)** forest plot of association between PDA and birth weight; **(D)** funnel plot for publication bias of the association between PDA and birth weight. PDA, patent ductus arteriosus.

**Figure 4:**
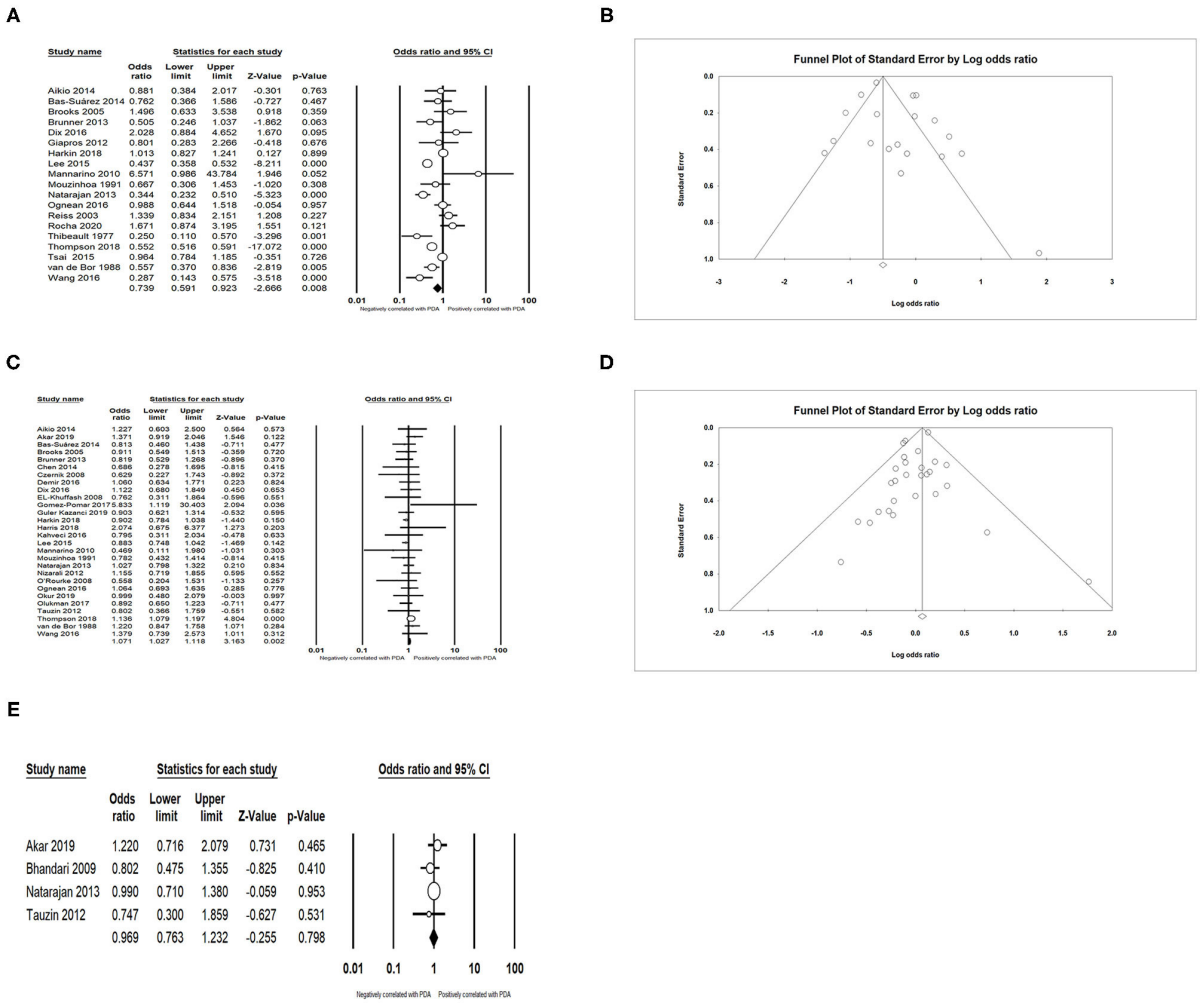
Results of neonatal characteristics in primary analysis. **(A)** Forest plot of association between PDA and small for gestational age; **(B)** funnel plot for publication bias of the association between PDA and small for gestational age; **(C)** forest plot of association between PDA and male gender (according to unadjusted data); **(D)** funnel plot for publication bias of the association between PDA and male gender (according to unadjusted data); **(E)** forest plot of association between PDA and male gender (according to adjusted data). PDA, patent ductus arteriosus.

We obtained an expected result that infants with lower GA (SMD = −0.698; 95% CI = −0.831 to −0.565; *I*^2^ = 93.794 %) (Figures 3A,B) and lower BW (SMD = −0.528; 95% CI = −0.669 to −0.387; *I*^2^ = 94.896 %) (Figures 3C,D) developed PDA more easily. The meta-analysis indicated that small for gestational age (SGA) could decrease the incidence of PDA (OR = 0.739; 95% CI = 0.591–0.923; *I*^2^ = 85.495%) (Figures 4A,B). Male infants were more likely to develop PDA compared to female infants in the light of the unadjusted data (OR = 1.071; 95% CI = 1.027–1.118; *I*^2^ = 26.025%) (Figures 4C,D), but pooled OR after adjustment for confounders showed no significant association between gender of infants and PDA (OR = 0.969; 95% CI = 0.763–1.232; *I*^2^ = 0%) (Figure 4E).

### Complications and Treatment

**Figure 5:**
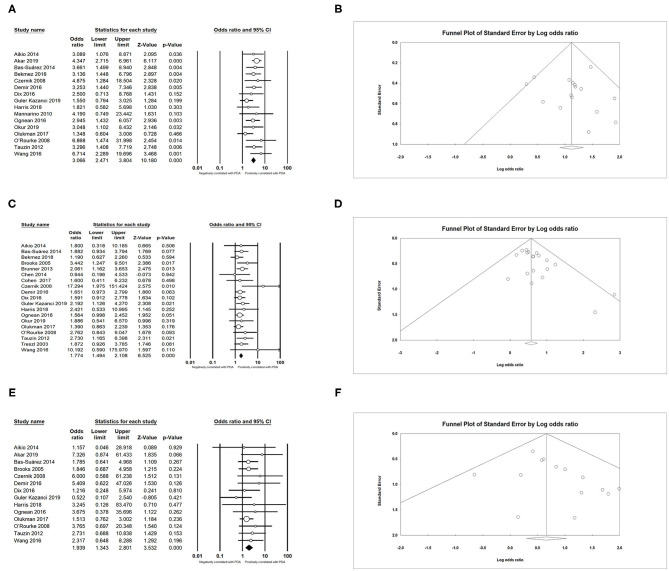
Results of complications in primary analysis. **(A)** Forest plot of association between PDA and bronchopulmonary dysplasia; **(B)** funnel plot for publication bias of the association between PDA and bronchopulmonary dysplasia; **(C)** forest plot of association between PDA and intraventricular hemorrhage; **(D)** funnel plot for publication bias of the association between PDA and intraventricular hemorrhage; **(E)** forest plot of association between PDA and necrotizing enterocolitis; **(F)** funnel plot for publication bias of the association between PDA and necrotizing enterocolitis. PDA, patent ductus arteriosus.

**Figure 6:**
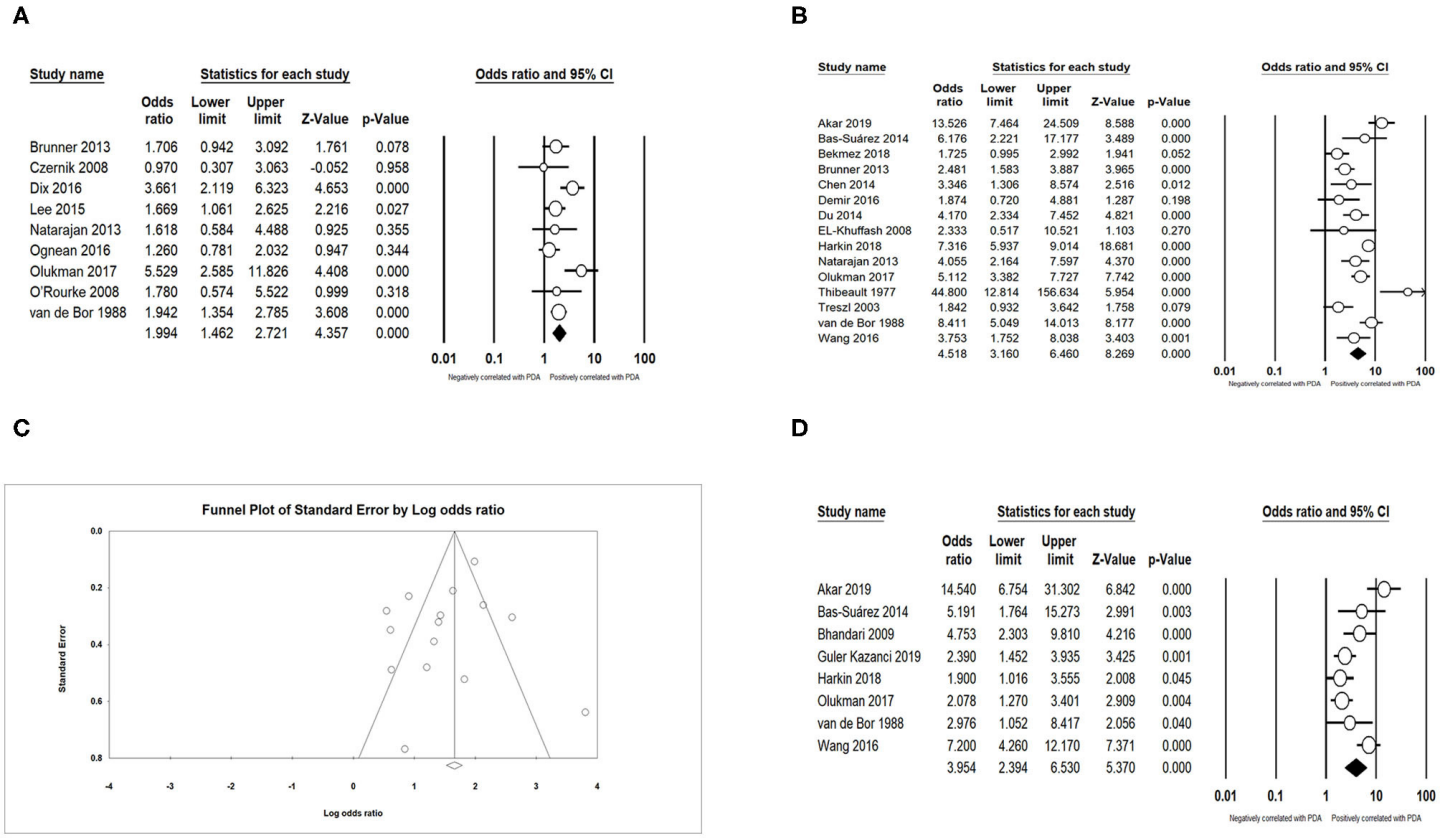
Results of complications in primary analysis. **(A)** Forest plot of association between PDA and sepsis; **(B)** forest plot of association between PDA and respiratory distress syndrome (according to unadjusted data); **(C)** funnel plot for publication bias of the association between PDA and respiratory distress syndrome; **(D)** forest plot of association between PDA and respiratory distress syndrome. PDA, patent ductus arteriosus.

**Figure 7:**
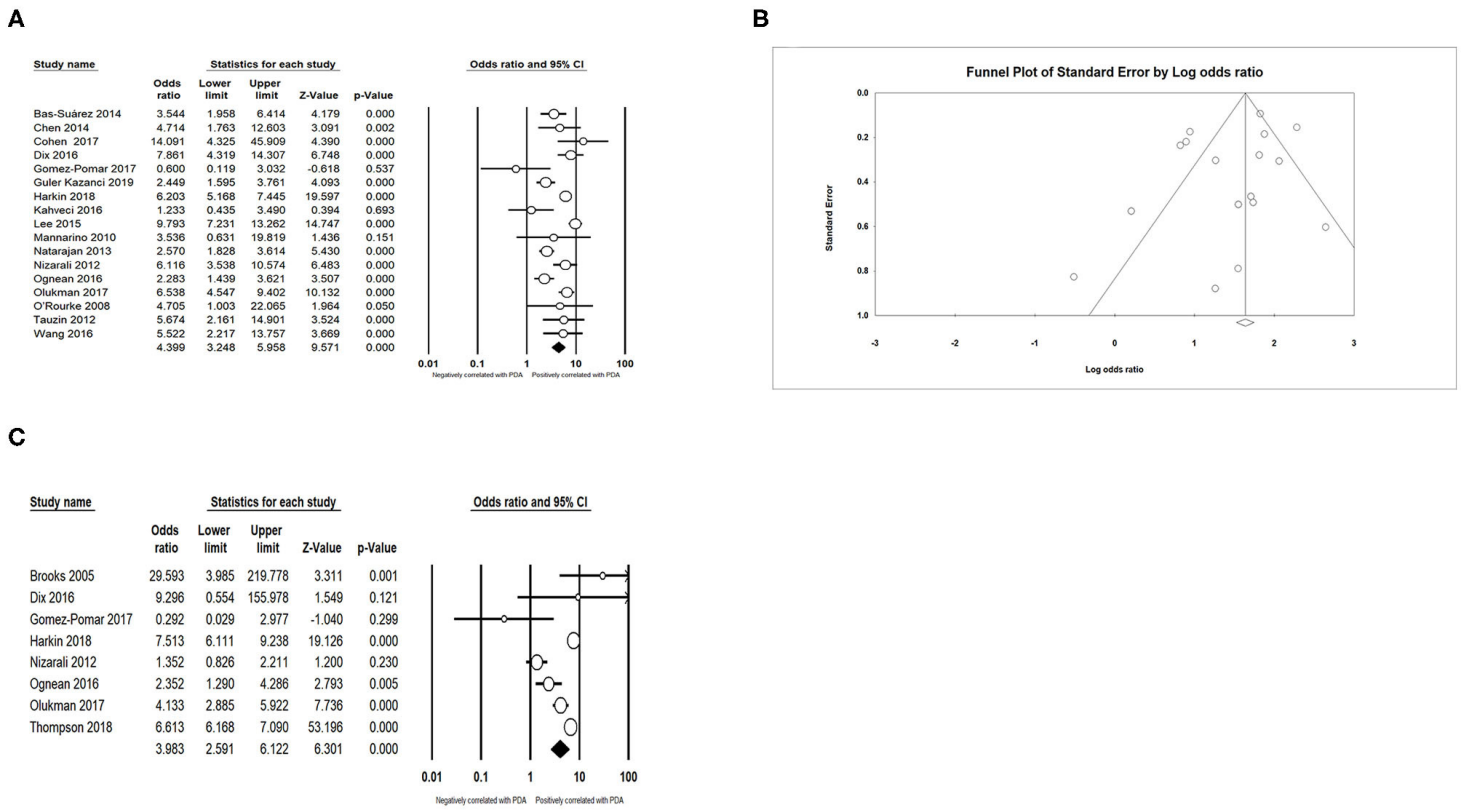
Results of treatment in primary analysis. **(A)** Forest plot of association between PDA and surfactant treatment; **(B)** funnel plot for publication bias of the association between PDA and surfactant treatment; **(C)** forest plot of association between PDA and ventilation. PDA, patent ductus arteriosus.

We found that PDA had a positive association with BPD (OR = 3.066; 95% CI = 2.471–3.804; *I*^2^ = 0%) (Figures 5A,B), IVH (OR = 1.774; 95% CI = 1.494–2.108; *I*^2^ = 0%) (Figures 5C,D), NEC (OR = 1.939; 95% CI = 1.343–2.801; *I*^2^ = 0%) (Figures 5E,F), and sepsis (OR = 1.994; 95% CI = 1.462–2.721; *I*^2^ = 54.919%) (Figure 6A). Both unadjusted (OR = 4.518; 95% CI = 3.160–6.460; *I*^2^ = 82.053%) (Figures 6B,C) and adjusted (OR = 3.954; 95% CI = 2.394–6.530; *I*^2^ = 78.081%) (Figure 6D) pooled data indicated that respiratory distress syndrome (RDS) was a clear risk factor of PDA. PDA was also significantly associated with surfactant treatment (OR = 4.399; 95% CI = 3.248–5.958; *I*^2^ = 80.788%) (Figures 7A,B) and ventilation (OR = 3.983; 95% CI = 2.591–6.122; *I*^2^ = 89.534%) (Figure 7C).

### Laboratory Examination

**Figure 8:**
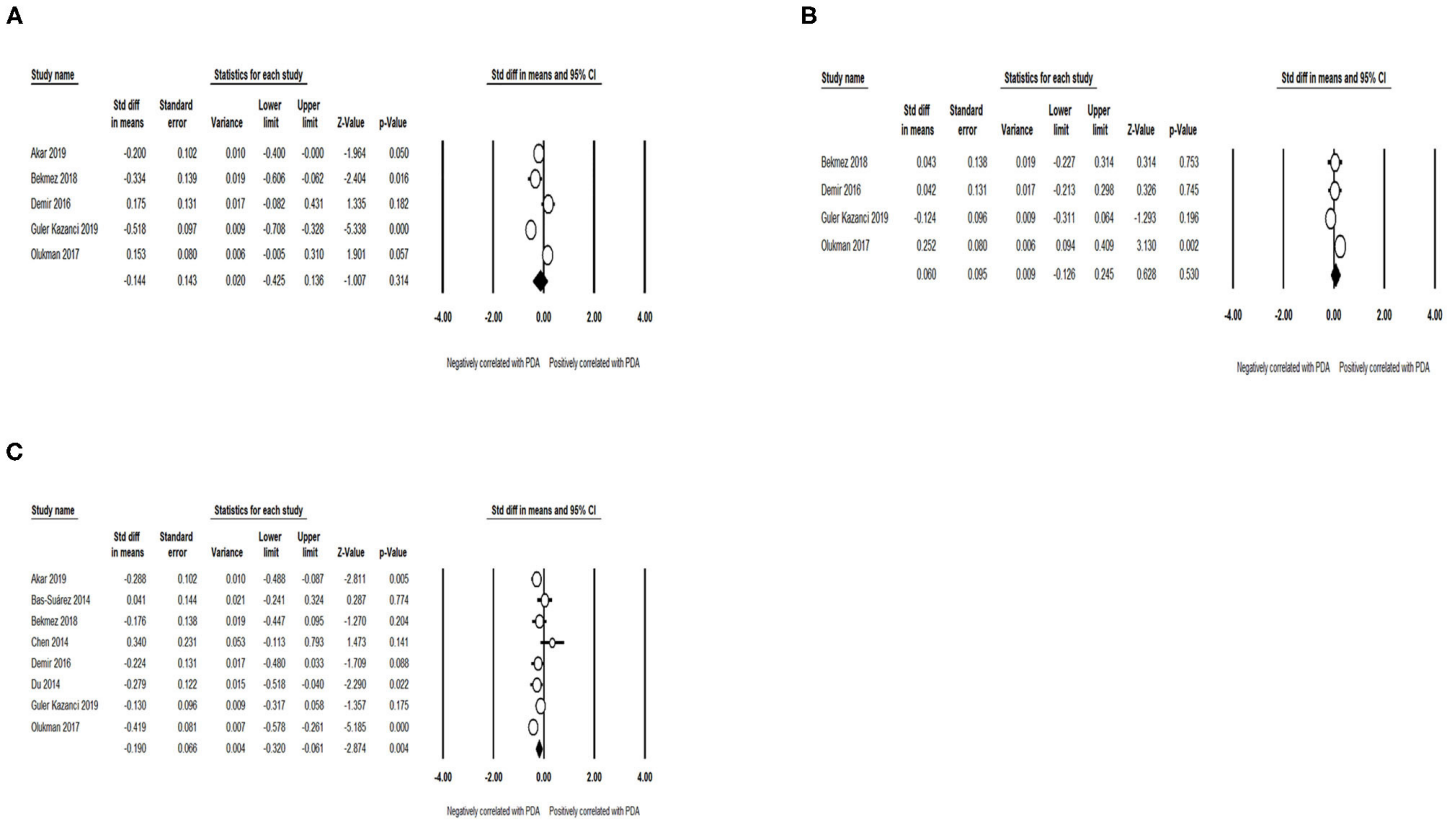
Results of laboratory examination in primary analysis. **(A)** Forest plot of association between PDA and mean platelet volume; **(B)** forest plot of association between PDA and platelet distribution width; **(C)** forest plot of association between patent ductus arteriosus PDA and platelet count. PDA, patent ductus arteriosus.

No significant association was discovered between the PDA and mean platelet volume (MPV) (SMD = −0.144; 95% CI = −0.425, 0.136; *I*^2^ = 89.011%) (Figure 8A) and platelet distribution width (PDW) (SMD = 0.060; 95% CI = −0.126 to 0.245; *I*^2^ = 67.558%) (Figure 8B). Meanwhile, pooled data demonstrated that low platelet count (SMD = −0.190; 95% CI = −0.320 to −0.061; *I*^2^ = 58.469%) (Figure 8C) improved the incidence of PDA among preterm infants.

### Sensitivity Analysis and Publication Bias

A sensitivity analysis was performed to evaluate the robustness of the conclusions by omitting each study sequentially and comparing the effects of the eliminations of each study on the pooled ORs or SMDs. We found that the exclusion of Thompson et al. (28) obviously changed the OR and 95% CI value for male gender to 0.951 (0.883–1.024), and the difference in OR and 95% CI value after the removal was statistically substantial. Therefore, we came to a revised conclusion that the gender of preterm infants had no effect on the occurrence of PDA (OR = 0.951; 95% CI = 0.883–1.024; *I*^2^ = 0%), and the detailed information is shown in Supplementary Table 4.

For every studied related factor mentioned in at least 10 articles, we not only drew a funnel plot, but also conducted Egger's test to evaluate its publication bias. If *P* < 0.10 in Egger's test, statistically significant publication bias was determined, and we carried out trim-and-fill computation to adjust the results. The results of the Egger's test suggested that the publication biases of IVH, BW, and GA were statistically significant (*P* < 0.10). However, the results of trim-and-fill computation showed that the identified biases did not interfere with interpretation of the results. The results of analysis concerning publication bias are recorded in Supplementary Table 5.

*P*-value in Egger's test of male gender was <0.10 in primary analysis. However, it turned to be non-significant after study exclusion of sensitivity analysis. Hence, we did not conduct trim-and-fill computation for male gender in the end, and we discussed the association between gender and PDA according to the results of sensitivity analysis.

## Discussion

It is a well-recognized fact that the incidence of PDA is inversely related to GA and BW of infants (15, 18, 29–34). The study of Koch et al. (35) showed that with each additional week of GA, the probability of spontaneous closure of the DA increased by 1.5 times. As noted above, the functional closure of DA is promoted by the increase of oxygen and the reduction of prostaglandins after birth. Nonetheless, preterm smooth muscle cells are more sensitive to circulating prostaglandins and nitric oxide, and even they can produce an increased amount of nitric oxide after birth (34, 36–39), while preterm DA's sensitivity to oxygen decreases, and preterm infants are often accompanied by hypoxemia (15, 17, 34). Besides, the term DA is more muscular and thicker-walled compared to the preterm DA, which is not sufficiently mature for the spontaneous closure after delivery (31, 38).

Our study confirms the results of prior meta-analysis (21), which supported a positive correlation between CA and PDA in preterm infants. On the one hand, CA is a definite risk factor for preterm birth (40, 41), and on the other hand, some cytokines regarding inflammatory process result in some hemodynamic changes (18, 21, 42). Interestingly, another meta-analysis on the same theme revealed that it was the confounders (differences in GA and BW) that considerably influenced the association between CA and PDA, and even CA played a protective role in the occurrence of PDA by facilitating lung maturation and diminishing the use of surfactant and mechanical ventilation (20, 43). It is a pity that the view cannot be proved in present analysis because we found only two studies showing pertinent data adjusted for confounders.

Although antenatal steroids has been reported to shield preterm infants from PDA (44) with an explanation that steroids appear to not only modify DA's sensitivity to prostaglandins, but also enhance the sensitivity of immature DA to oxygen, affect the synthesis of prostaglandins, and decrease the incidence of neonatal RDS (15, 17, 18), this affiliation was not found in this meta-analysis in accordance with both unadjusted and adjusted data. The following reasons probably can explain the surprising result. First, we did not distinguish infants with full course and partial course of treatment in the data synthesis process. Second, the number of included articles was not sufficient, and some valuable articles might be omitted, especially only three articles presenting adjusted data. Third, none of the included articles took the relationship between PDA and antenatal steroids as the main research purpose, and most of these articles had a retrospective design, which would have an impact on the results of these studies. Besides, Shelton et al. found that among infants born at <26 weeks, antenatal steroids could constrict DA apparently only when prostaglandin production had been eliminated. Although they did not observe the phenomenon among infants born at 26–28 weeks, they speculated that the effects of antenatal steroids might be different depending on the GA of infants (45).

Previous literature has indicated that PROM (46) and preeclampsia (47, 48) are predictors for preterm birth, so it seems quite reasonable that they are also risk factors for PDA. Surprisingly, our results manifest that they unexpectedly play a non-promotive role in the occurrence of PDA. In fact, the seemingly irrational results can still be interpreted by the theory that PROM (49) and preeclampsia (18) can exert maturational effects on pulmonary function, and moreover, mothers complicated by these conditions have a higher possibility to obtain more efficient management and more adequate treatment, which prolongs GA and prevents PDA after birth (50, 51).

Our results are compatible with the findings of Villamor-Martinez et al., who pointed out that infants diagnosed with SGA were characterized by lower risk for PDA in comparison with those in control group. In their meta-analysis, a noteworthy difference in GA owing to fewer “indicated deliveries” in SGA group than in control group was detected, which could account for the negative association between SGA and PDA (23), and Wang et al. also proposed this hypothesis (19). Hammoud et al. reported male gender was a risk factor for PDA (52). However, both pooled unadjusted results after sensitivity analysis and pooled adjusted results in our study show no association between gender and PDA.

We corroborate that infants with PDA are at a greater risk of various neonatal diseases. Left-to-right shunting across the DA results in pulmonary overcirculation and decreased systemic perfusion. “Diastolic steal” through the DA to the lungs reduces the blood supply to other organs, escalating the rate of IVH and NEC (18, 49, 53–58). The mechanism of RDS causing PDA is mainly related to hypoxemia, and pulmonary hyperperfusion resulting from PDA impairs surfactant production in the alveolar cells, which means the relationship between PDA and RDS is complex and bidirectional (15, 18, 23, 49). PDA can promote an interstitial and alveolar pulmonary edema and decreased lung compliance, and in turn, it gives rise to higher ventilator settings and prolonged ventilation duration, which has been implicated in the pathogenesis of BPD (18, 49, 54, 59–63). In recent studies, Clyman et al. found that exposure to PDA increased risk of BPD only when the infants required at least 10 days of intubation, and the incidence of BPD (grades 2 and 3) was low in infants requiring less ventilatory support, regardless of whether PDA constricted or remained open for several weeks (64, 65). The positive correlation between PDA and sepsis can be attributed to elevated prostaglandins in infected infants (18, 49, 66).

Given the tight relationship between PDA and RDS, it can be readily understood that infants with PDA are more likely to receive surfactant and ventilation administration (18, 67). What's more, Nizarali et al. proposed that a significant drop in pulmonary vascular resistance after surfactant administration would cause a larger shunt through DA, indicating that the use of surfactant would aggravate PDA (17).

A number of studies have suggested the compelling relationship between low platelet count and PDA (14, 22, 68–70), and our study further substantiates these results. Echtler et al. identified that platelets were recruited to contract DA within minutes after birth, initiate thrombotic occlusion of DA, and support subsequent anatomical reorganization (68). MPV and PDW are non-significant in predicting PDA in our results despite the statement that they are markers reflecting platelet activity (33, 71).

We conducted an extra analysis focusing only on the included articles in the last 10 years because some of included articles are so outdated that they probably affect the stability of our conclusions as a consequence of the fact that there have been many changes in neonatal intensive care unit such as higher conservative management rates and promotion of advanced prevention and treatment measures of PDA in developing countries. However, the pooled results of articles in the last 10 years were not statistically different from original results for each factor except PROM, and its OR and 95% CI values changed to 0.713 (0.574–0.884), which meant PROM could decrease the risk of PDA. But this change will not contradict our previous explanation, for PROM still plays a non-promotive role in the occurrence of PDA according to the results of included articles in last 10 years.

This meta-analysis has several limitations. First, all the included articles were observational studies, and most of them had a retrospective design, which could arouse inherent bias. Second, there was a significant variability among studies with regard to the definition of PDA, baseline characteristics of neonates, and included confounders. Third, some valuable factors, such as B-type natriuretic peptide level and fluid intake, could not be analyzed in this meta-analysis because of insufficient data. Fourth, most included articles did not make a multivariate regression analysis, so for most factors, we cannot determine their independent effects for PDA.

## Conclusion

To our best knowledge, this is the first comprehensive meta-analysis taking into account multiple related factors of PDA, and a total of 18 potential related factors were examined. After a series of data analysis, the final results revealed that CA, lower GA, lower BW, BPD, IVH, NEC, RDS, sepsis, surfactant treatment, ventilation, and lower platelet count were positively related to PDA, whereas SGA exerted a converse effect among preterm infants. Additionally, we also found that PROM, preeclampsia, antenatal steroids, male gender, MPV, and PDW had no statistically significant link with PDA. Our findings can be helpful to design prevention strategies and management standards for PDA. Also, future large prospective studies are strongly demanded to validate the accuracy and reliability of the conclusions.

## Data Availability Statement

The raw data supporting the conclusions of this article will be made available by the authors, without undue reservation. Requests to access these datasets should be directed to Chang Liu, 1113943180@qq.com.

## Author Contributions

CL proposed the idea and designed the whole research plan. CL and XZ conducted the database search and extracted the data. CL and DL performed the data analysis and prepared the figures and the tables. CL wrote the manuscript. All authors reviewed the manuscript.

## Conflict of Interest

The authors declare that the research was conducted in the absence of any commercial or financial relationships that could be construed as a potential conflict of interest.
