# Supplementary material for: Related Factors of Patent Ductus Arteriosus in Preterm Infants: A Systematic Review and Meta-Analysis
Source: Front Pediatr. 2021 Jan 5;8:605879. doi: 10.3389/fped.2020.605879 (PMC7813817; doi:10.3389/fped.2020.605879)
Supplement: Supplementary file 1 [file Data_Sheet_1.docx]

Supplementary Material

- **Related factors of Patent Ductus Arteriosus in Preterm Infants: A Systematic Review and Meta-Analysis**

**Chang Liu^1,2,3^, Xingwang Zhu^1,2,3^, Dinggang Li^1,2,3^, Yuan Shi^1,2,3,4*^**

^1^Department of Neonatology, Children's Hospital of Chongqing Medical University, Chongqing, China.

^2^National Clinical Research Center for Child Health and Disorders, Chongqing, China.

^3^Ministry of Education Key Laboratory of Child Development and Disorders, Chongqing, China.

^4^Chongqing Key Laboratory of Pediatrics, Chongqing, China.

***Correspondence:**Yuan Shi
shiyuan@hospital.cqmu.edu.cn

1. **Supplementary Tables**

**Supplementary Table 1.**Characteristics of all included studies

| First author | Year | Country/ Region | Prosp/ Retro | Study type (ca-co/cohort) | Infants(n) | Mean birth weight(g) | Mean gestational age(weeks) | Definition of PDA | PDA assessment, time of life | Aim of study |
| --- | --- | --- | --- | --- | --- | --- | --- | --- | --- | --- |
| Aikio | 2014 | Finland | Retro | cohort | 190 | 1251 | 28.7 | hsPDA | Day 2-5 | To determine whether early paracetamol therapy was associated with decreased incidence of PDA without adverse events. |
| Akar | 2019 | Turkey | Retro | cohort | 389 | 1064 | 29.4 | hsPDA | 48-96h | To evaluate whether there is an association between the platelet mass and patent ductus arteriosusclosure in premature newborns. |
| Arayici | 2014 | Turkey | Retro | ca-co | 281 | 1173 | 28.9 | Treat | NA | To determine the effect of histological CA on the short-term outcome of preterm infants. |
| Bas-Suárez | 2014 | Spain | Retro | cohort | 194 | 1008 | 27.9 | hsPDA | day 2-4 | To investigate the association between platelet count nadir within the first 7 days of life and the rate of hemodynamically significant PDA, as well as the rate of response to the treatment with cyclooxygenase inhibitors. |
| Bekmez | 2018 | Turkey | Retro | cohort | 212 | 1238 | 29.0 | hsPDA | 48-72h | To evaluate whether RPR would have a role in the diagnosis and/or prediction of pharmacological closure of PDA. |
| First author | Year | Country/ Region | Prosp/ Retro | Study type (ca-co/cohort) | Infants(n) | Mean birth weight(g) | Mean gestational age(weeks) | Definition of PDA | PDA assessment, time of life | Aim of study |
| Bhandari | 2009 | USA | Retro | ca-co | 864 | 1638 | 31.1 | Echocardiogram | NA | To quantify the contribution of genetic factors to the variance in liability for patent ductus arteriosus in premature newborns. |
| Brooks | 2005 | Australia | Prosp | cohort | 252 | 907 | 26.0 | hsPDA | NA | To test the hypothesis that outcomes of infants with persistent patent ductus arteriosus were no worse than those of infants with no significant duct or a duct that closed after medical treatment |
| Brunner | 2013 | Austria | Prosp | cohort | 322 | 1159 | 28.4 | Echocardiogram | Day 2 for infants with severe RDS, and day 4-7 for the others | To assess the risk for intraventricular hemorrhage in very low birth weight preterm infants with patent ductus arteriosus and low platelet count with treatment with cyclooxygenase inhibitors. |
| Chen | 2014 | Taiwan, China | Retro | ca-co | 77 | 1153 | 29.5 | Echocardiogram with clinical signs and a need for treatment | NA | To assess the influence of hemoglobin and perinatal factors on PDA in very low birth weight infants |
| Cohen | 2017 | Netherlands | Retro | ca-co | 72 | 958 | 28.6 | hsPDA and treat | Within the first week | To assess the effect of hsPDA on cerebral oxygenation in preterm SGA neonates compared to AGA peers |
| First author | Year | Country/ Region | Prosp/ Retro | Study type (ca-co/cohort) | Infants(n) | Mean birth weight(g) | Mean gestational age(weeks) | Definition of PDA | PDA assessment, time of life | Aim of study |
| Czernik | 2008 | Germany | Prosp | cohort | 67 | 866 | 25.7 | Echocardiogram and need for ventilatory support. | 24-48h | To determine whether BNP can predict the need for PDA intervention |
| Demir | 2016 | Turkey | Retro | ca-co | 235 | 1524 | 28.9 | hsPDA | Day 3-5 | To evaluate whether or not platelet mass contributes to closure of patent ductus arteriosus in premature newborns. |
| Dix | 2016 | Netherlands | Prosp | cohort | 398 | 1108 | 28.3 | hsPDA | Day 2,4 and 6 | To determine the relationship between echocardiographic parameters, cerebral oxygenation and anhsPDA in preterm infants. |

| Du | 2014 | China | Retro | ca-co | 136 | 1531 | 30.6 | hsPDA | Within day 3 | To investigate the risk factors for the occurrence of patent ductus arteriosus (PDA) and to provide a clinical basis for reducing the occurrence of PDA in early preterm infants |
| --- | --- | --- | --- | --- | --- | --- | --- | --- | --- | --- |
| EL-Khuffash | 2008 | Ireland | Prosp | cohort | 80 | 1036 | 28.2 | Echocardiogram | 12-48h | To evaluate the effect of patent ductus arteriosus on the myocardium by measuring levels of cardiac troponin T, a marker of ischemic myocardial damage. |
| García-Muñoz | 2014 | Spain | Retro | cohort | 8330 | 1086 | 28.5 | Echocardiogram and need for treatment | NA | To determine the incidence of clinical chorioamnionitis and its relationship to morbidity and mortality among very-low-birth-weight infants |
| First author | Year | Country/ Region | Prosp/ Retro | Study type (ca-co/cohort) | Infants(n) | Mean birth weight(g) | Mean gestational age(weeks) | Definition of PDA | PDA assessment, time of life | Aim of study |
| Giapros | 2012 | Greece | Retro | cohort | 210 | 1136 | 28.5 | Treat | NA | To study comparatively morbidity and mortality in SGA and AGA neonates born with low gestational age. |
| Gomez-Pomar | 2017 | USA | Prosp | cohort | 31 | 844 | 26.2 | Echocardiogram | NA | To assess the value of continuous high resolution ∆PI monitoring in the diagnosis of PDA. |
| GulerKazanci | 2019 | Turkey | Retro | ca-co | 481 | 1068 | 28.0 | hsPDA | 48-96h | To assess the utility of early postnatal platelet indices in the prediction of hemodynamically significant patent ductus arteriosus and its response to pharmacological treatment in preterm infants. |
| Härkin | 2018 | Finland | Prosp | cohort | 3668 | 1244 | 29.1 | Treat | NA | To evaluate the predictive factors for the development of hemodynamically significant PDA in preterm infants and to study the morbidities associated with the treatment ofPDA during the first hospitalization. |
| Harris | 2018 | New Zealand | Prosp | cohort | 51 | 1094 | 27.8 | hsPDA | Day 3 | To investigate factors affecting N-terminal pro-B-type natriuretic peptide (NTproBNP) in preterm infants and the ability ofNTproBNP to predict hemodynamically significant patent ductus arteriosus. |
| Huang | 2015 | Taiwan, China | Retro | cohort | 5718 | 1161 | 29.3 | Treat | NA | To evaluate the association between maternal preeclampsia and ROP. |
| First author | Year | Country/ Region | Prosp/ Retro | Study type (ca-co/cohort) | Infants(n) | Mean birth weight(g) | Mean gestational age(weeks) | Definition of PDA | PDA assessment, time of life | Aim of study |
| Kachikis | 2019 | USA | Retro | cohort | 1729 | 1407 | 29.8 | Treat | NA | To determine if PPROM imparts a differentially greater risk for neonatal BPD than sPTL. |
| Kahveci | 2016 | Turkey | Prosp | cohort | 72 | 1852 | 30.0 | hsPDA | 48-96h | To investigate the diagnostic significance of serum ischemia modified albumin levels as a screening tool for hsPDA, and its relation to the severity of the disease in the neonates |
| Lee | 2015 | South Korea | Prosp | cohort | 2254 | 1069 | 28.3 | Clinical symptoms | NA | To investigate current therapeutic strategies for patent ductus arteriosus in very-low-birth-weight infants in Korea |
| Mannarino | 2010 | Italy | Prosp | cohort | 36 | 1338 | 30.8 | Echocardiogram | Within day 3 | To evaluate and compare cardiovascular adaptation of36 preterm and 34 fullterm newborns |
| Mouzinhoa | 1991 | USA | Prosp | cohort | 189 | 1047 | 28.0 | hsPDA | NA | NA |
| Natarajan | 2013 | USA | Prosp | cohort | 968 | 761 | 25.8 | hsPDA | NA | To evaluate the relationship, if any, between blood spot TGF-β on day 3 and day 7 of life and patent ductus arteriosus in extremely low birth weight infants |
| Nizarali | 2012 | Portugal | Retro | ca-co | 318 | 1139 | 29.0 | Echocardiogram | NA | Identification of perinatal risk factors associated with PDA in premature or very low birth weight infants. |
| First author | Year | Country/ Region | Prosp/ Retro | Study type (ca-co/cohort) | Infants(n) | Mean birth weight(g) | Mean gestational age(weeks) | Definition of PDA | PDA assessment, time of life | Aim of study |
| O’Rourke | 2008 | Ireland | Prosp | cohort | 87 | 1159 | 28.5 | hsPDA | 12h and day 3 | To evaluate the effect of serial echocardiography performed by a neonatologist and early targeted medical PDA treatment and compared to historical controls |
| Ognean | 2016 | Romania | ? | cohort | 340 | 1385 | 30.0 | Echocardiogram | After day 7 | To evaluate the association between the presence of PDA and the severity of clinical condition at birth in critically ill preterm infants, with gestational ages ≤ 32 weeks and severe respiratory distress |
| Okur | 2019 | Turkey | Prosp | cohort | 119 | 1068 | 28.6 | hsPDA | Within day 3 | To determine the usefulness of high lactate levels as a marker in hemodynamically significant patent ductus arteriosus, which may lead to tissue perfusion defects. |
| Olukman | 2017 | Turkey | Retro | ca-co | 824 | 1193 | 29.5 | hsPDA | Within day 4 | To assess whether a relationship exists between the occurrence and/or closure of hemodynamically significant ductus arteriosus and platelet parameters (platelet count, circulating platelet mass, mean platelet volume, platelet distribution width) in preterm newborns. |
| First author | Year | Country/ Region | Prosp/ Retro | Study type (ca-co/cohort) | Infants(n) | Mean birth weight(g) | Mean gestational age(weeks) | Definition of PDA | PDA assessment, time of life | Aim of study |
| Reiss | 2003 | Germany | Prosp | cohort | 1365 | 1196 | 28.8 | NA | NA | To evaluate the impact of being born small for gestational age on neonatal mortality and neonatal pulmonary morbidity in preterm infants <32 weeks of gestation. |
| Rocha | 2020 | Portugal | Retro | cohort | 494 | 963 | 27.6 | hsPDA | 24-72h | To assess the association between low birth weight and BPD in preterm infants |
| Seliga-Siwecka | 2013 | Poland | Prosp | cohort | 383 | 1338 | 29.2 | Echocardiogram | NA | To 1) identify genital tract colonization of women with chorioamnionitis and 2) evaluate the link between specific bacterial colonization of the maternal genital tract, and neonatal outcome of extremely premature infants exposed to chorioamnionitis |
| Tauzin | 2012 | France | Retro | ca-co | 137 | 1065 | 27.8 | hsPDA | Beyond day 3 | To investigate the possible effect of persistent patent ductus in VLBW infants. |
| Thibeault | 1977 | USA | ? | cohort | 144 | 1177 | 29.6 | Clinical examination or retrograde aortography | After 24h | To answer the question: Is there any relationship between RDS and patency of the ductus arteriosus in preterm infants, that is, do preterm infants without the RDS have early functional closure of the ductus arteriosus? |
| First author | Year | Country/ Region | Prosp/ Retro | Study type (ca-co/cohort) | Infants(n) | Mean birth weight(g) | Mean gestational age(weeks) | Definition of PDA | PDA assessment, time of life | Aim of study |
| Thompson | 2018 | USA | Retro | cohort | 43576 | 1101 | 28.5 | Treat | NA | To evaluate the association between furosemide exposure and patent ductus arteriosus in a large, contemporary cohort of hospitalized infants with very low birth weight. |
| Treszl | 2003 | Hungary | Retro | cohort | 159 | 1185 | 29.2 | Echocardiogram or clinical signs | Within day 6 | NA |
| Tsai | 2015 | Taiwan, China | Retro | cohort | 1680 | 1052 | 28.3 | NA | NA | To evaluate the impact of small for gestational age on mortality and morbidity in very low birth weight infants |
| van de Bor | 1988 | Netherlands | Prosp | cohort | 1252 | 1270 | 30.5 | hsPDA | NA | To establish the predictive value of various perinatal factors frequently associated with the occurrence of PDA. |
| Wang | 2016 | China | Retro | ca-co | 194 | 1217 | 29.8 | hsPDA | NA | To investigate the factors influencing the prognosis of patent ductus arteriosus in very low birth weight infants. |
| Yen | 2013 | Taiwan, China | Retro | cohort | 8653 | 1183 | 29.3 | Treat | NA | To test whether or not preeclampsia is associated with development of BPD in a cohort of premature infants. |
| First author | Year | Country/ Region | Prosp/ Retro | Study type (ca-co/cohort) | Infants(n) | Mean birth weight(g) | Mean gestational age(weeks) | Definition of PDA | PDA assessment, time of life | Aim of study |
| Yum | 2018 | South Korea | Retro | cohort | 188 | 1269 | 29.3 | Treat | NA | To evaluate the association between the presence of histologic chorioamnionitis and development of pulmonary hypertension during neonatal intensive care unit stay. |

Abbreviations: PDA, patent ductus arteriosus; Prosp, prospective; Retro, retrospective; ?, unknown; ca-co, case-control study; cohort, cohort study; hsPDA, hemodynamically significant PDA; Treat, medically or surgically treated PDA; NA (definition of PDA), no diagnostic criteria mentioned; Echocardiogram, PDA defined by echocardiogram; NA (PDA assessment, time of life), no concrete time mentioned; CA, chorioamnionitis; RPR, red cell distribution width-to-platelet ratio; SGA, small for gestational age; AGA, appropriate for gestational age; BNP, B-type natriuretic peptide; ∆PI, change value of perfusion index; ROP, retinopathy of prematurity; NA, no aims mentioned; PPROM, preterm premature rupture of membranes; BPD, bronchopulmonary dysplasia; sPTL, spontaneous preterm labor; TGF-β,transforming growth factor-β; VLBW, very low birth weight; RDS, respiratory distress syndrome.

**Supplementary Table 2.** Details of Newcastle-Ottawa Quality assessment.

| First author | Year | Selection | Comparability | Outcome /Exposure | Total | Reason for downgrade |
| --- | --- | --- | --- | --- | --- | --- |
| Aikio | 2014 | 4 | 0 | 3 | 7 | No adjustment for confounders |
| Akar | 2019 | 4 | 2 | 3 | 9 |  |
| Arayici | 2014 | 4 | 0 | 3 | 7 | No adjustment for confounders |
| Bas-Suárez | 2014 | 4 | 2 | 3 | 9 |  |
| Bekmez | 2018 | 4 | 0 | 3 | 7 | No adjustment for confounders |
| Bhandari | 2009 | 3 | 2 | 3 | 8 | All infants are twins |
| Brooks | 2005 | 4 | 0 | 3 | 7 | No adjustment for confounders |
| Brunner | 2013 | 4 | 0 | 3 | 7 | No adjustment for confounders |
| Chen | 2014 | 4 | 0 | 3 | 7 | No adjustment for confounders |
| Cohen | 2017 | 4 | 0 | 3 | 7 | No adjustment for confounders |
| Czernik | 2008 | 4 | 0 | 3 | 7 | Adjusted data not shown |
| Demir | 2016 | 4 | 2 | 3 | 9 |  |
| Dix | 2016 | 4 | 0 | 3 | 7 | No adjustment for confounders |
| Du | 2014 | 4 | 2 | 3 | 9 |  |
| EL-Khuffash | 2008 | 4 | 0 | 3 | 7 | No adjustment for confounders |
| García-Muñoz | 2014 | 4 | 2 | 3 | 9 |  |
| Giapros | 2012 | 4 | 0 | 3 | 7 | No adjustment for confounders |
| Gomez-Pomar | 2017 | 3 | 0 | 3 | 6 | No adjustment for confounders; PDA not clearly defined |
| GulerKazanci | 2019 | 4 | 2 | 3 | 9 |  |
| Härkin | 2018 | 4 | 2 | 3 | 9 |  |
| Harris | 2018 | 4 | 0 | 3 | 7 | No adjustment for confounders |
| Huang | 2015 | 4 | 0 | 3 | 7 | No adjustment for confounders |
| Kachikis | 2019 | 4 | 0 | 3 | 7 | No adjustment for confounders |
| Kahveci | 2016 | 4 | 0 | 3 | 7 | No adjustment for confounders |
| Lee | 2015 | 4 | 0 | 3 | 7 | No adjustment for confounders |
| Mannarino | 2010 | 4 | 0 | 3 | 7 | No adjustment for confounders |
| Mouzinhoa | 1991 | 4 | 0 | 3 | 7 | No adjustment for confounders |
| Natarajan | 2013 | 4 | 2 | 3 | 9 |  |
| Nizarali | 2012 | 3 | 2 | 3 | 8 | PDA not clearly defined |
| O’Rourke | 2008 | 4 | 0 | 3 | 7 | No adjustment for confounders |
| Ognean | 2016 | 3 | 0 | 3 | 6 | No adjustment for confounders; PDA not clearly defined |
| Okur | 2019 | 4 | 0 | 3 | 7 | No adjustment for confounders |
| Olukman | 2017 | 4 | 2 | 3 | 9 |  |
| Reiss | 2003 | 4 | 0 | 2 | 6 | No adjustment for confounders; PDA not defined |
| Rocha | 2020 | 4 | 1 | 3 | 8 | Adjusted data on PDA not shown |
| Seliga-Siwecka | 2013 | 4 | 2 | 3 | 9 |  |
| Tauzin | 2012 | 4 | 2 | 3 | 9 |  |
| Thibeault | 1977 | 3 | 0 | 3 | 6 | No adjustment for confounders; PDA not clearly defined |
| Thompson | 2018 | 4 | 1 | 3 | 8 | Just showing the adjusted data of only one factor |
| Treszl | 2003 | 4 | 2 | 3 | 9 |  |
| Tsai | 2015 | 3 | 0 | 3 | 6 | Needed adjusted data not shown; PDA not defined |
| van de Bor | 1988 | 4 | 2 | 3 | 9 |  |
| Wang | 2016 | 4 | 0 | 3 | 7 | No adjustment for confounders |
| Yen | 2013 | 4 | 0 | 3 | 7 | No adjustment for confounders |
| Yum | 2018 | 4 | 0 | 3 | 7 | No adjustment for confounders |

Abbreviation: PDA, patent ductus arteriosus.

**Supplementary Table 3.**The primary pooled results of the association between related factors and PDA.

| Related factor | Number of studies | Primary pooled result | | | P-value  (Egger's test) | Heterogeneity test | | |
| --- | --- | --- | --- | --- | --- | --- | --- | --- |
|  |  | OR/SMD | 95% CI | P-value |  | P-value | I² (%) | Meta analytical model |
| Antenatal steroids | 23 | 0.931 | 0.822, 1.054 | 0.257 | 0.709 | 0.041 | 36.655 | Random |
| Antenatal steroids (adjusted data) | 3 | 0.727 | 0.479, 1.102 | 0.133 | NA | 0.052 | 66.286 | Random |
| BPD | 16 | 3.066 | 2.471, 3.804 | 0.000 | 0.780 | 0.456 | 0.000 | Fixed |
| CA | 11 | 1.317 | 1.081, 1.604 | 0.006 | 0.246 | 0.072 | 41.578 | Random |
| IVH | 19 | 1.774 | 1.494, 2.108 | 0.000 | 0.011 | 0.770 | 0.000 | Fixed |
| Male gender | 28 | 1.071 | 1.027, 1.118 | 0.002 | 0.074 | 0.105 | 26.025 | Fixed |
| Male gender (adjusted data) | 4 | 0.969 | 0.763, 1.232 | 0.798 | NA | 0.672 | 0.000 | Fixed |
| NEC | 14 | 1.939 | 1.343, 2.801 | 0.000 | 0.109 | 0.831 | 0.000 | Fixed |
| Preeclampsia | 8 | 0.890 | 0.677, 1.171 | 0.406 | NA | 0.001 | 72.730 | Random |
| PROM | 8 | 0.757 | 0.568, 1.009 | 0.057 | NA | 0.065 | 47.358 | Random |
| RDS | 15 | 4.518 | 3.160, 6.460 | 0.000 | 0.269 | 0.000 | 82.053 | Random |
| RDS (adjusted data) | 8 | 3.954 | 2.394, 6.530 | 0.000 | NA | 0.000 | 78.081 | Random |
| Sepsis | 9 | 1.994 | 1.462, 2.721 | 0.000 | NA | 0.023 | 54.919 | Random |
| SGA | 19 | 0.739 | 0.591, 0.923 | 0.008 | 0.123 | 0.000 | 85.495 | Random |
| Surfactant treatment | 17 | 4.399 | 3.248, 5.958 | 0.000 | 0.234 | 0.000 | 80.788 | Random |
| Ventilation | 8 | 3.983 | 2.591, 6.122 | 0.000 | NA | 0.000 | 89.534 | Random |
| BW | 30 | -0.528 | -0.669, -0.387 | 0.000 | 0.000 | 0.000 | 94.896 | Random |
| GA | 29 | -0.698 | -0.831, -0.565 | 0.000 | 0.007 | 0.000 | 93.794 | Random |
| MPV | 5 | -0.144 | -0.425, 0.136 | 0.314 | NA | 0.000 | 89.011 | Random |
| Platelet count | 8 | -0.190 | -0.320, -0.061 | 0.004 | NA | 0.018 | 58.469 | Random |
| PDW | 4 | 0.060 | -0.126, 0.245 | 0.530 | NA | 0.026 | 67.558 | Random |

Abbreviations: OR, odds ratio; SMD, standard mean difference; CI, confidence intervals; NA, included studies less than 10, thus unable to carry out Egger’s test; BPD, bronchopulmonary dysplasia; CA, chorioamnionitis; IVH, intraventricular hemorrhage; NEC, necrotizing enterocolitis; PROM, premature rupture of membranes; RDS, respiratory distress syndrome; SGA, small for gestational age; BW, birth weight; GA, gestational age; MPV, mean platelet volume; PDW, platelet distribution width.

**Supplementary Table 4.** Pooled results before and after sensitivity analysis.

| Related factor | Before sensitivity analysis | | | | | The excluded study | After sensitivity analysis | | | | |
| --- | --- | --- | --- | --- | --- | --- | --- | --- | --- | --- | --- |
|  | Pooled OR/SMD | 95% CI | P-value (pooled results) | P-value (heterogeneity test） | I² (%) |  | Pooled OR/SMD | 95% CI | P-value (pooled results) | P-value (heterogeneity test） | I² (%) |
| Male gender | 1.071 | 1.027, 1.118 | 0.002 | 0.105 | 26.025 | Thompson 2018 | 0.951 | 0.883, 1.024 | 0.183 | 0.708 | 0.000 |

Abbreviations: OR, odds ratio; SMD, standard mean difference; CI, confidence intervals.

**Supplementary Table 5.**Pooled results of related factors with significant publication bias before and after trim-and-fill computation.

| Related factor | P-value (Egger's Test) | Primary pooled results | | Results adjusted after trim-and-fill computation | | Whether interfere with interpretation of the result |
| --- | --- | --- | --- | --- | --- | --- |
|  |  | Pooled OR/SMD | 95% CI | Pooled OR/SMD | 95% CI |  |
| IVH | 0.011 | 1.774 | 1.494, 2.108 | 1.647 | 1.398, 1.941 | No |
| BW | 0.000 | -0.528 | -0.669, -0.387 | -0.932 | -1.076, -0.789 | No |
| GA | 0.007 | -0.698 | -0.831, -0.565 | -0.948 | -1.086, -0.810 | No |

Abbreviations: OR, odds ratio; SMD, standard mean difference; CI, confidence intervals; IVH, intraventricular hemorrhage; BW, birth weight; GA, gestational age.
